# The Phosphoproteomic Response of Okra (*Abelmoschus esculentus* L.) Seedlings to Salt Stress

**DOI:** 10.3390/ijms20061262

**Published:** 2019-03-13

**Authors:** Chenliang Yu, Qinqfei Wu, Chendong Sun, Mengling Tang, Junwei Sun, Yihua Zhan

**Affiliations:** 1 Institute of Agricultural Equipment, Zhejiang Academy of Agricultural Sciences, Hangzhou 310021, China; 21007030@zju.edu.cn; 2 State Key Laboratory of Plant Physiology and Biochemistry, College of Life Sciences, Zhejiang University, Hangzhou 310058, China; feiqw1234@163.com; 3 The Key Laboratory for Quality Improvement of Agricultural Products of Zhejiang Province, School of Agriculture and Food Science, Zhejiang A&F University, Linan, Hangzhou 311300, China; SCD20180013@163.com (C.S.); tmling789@163.com (M.T.); 4 College of modern science and technology, China Jiliang University, Hangzhou 310018, China; juville@cjlu.edu.cn

**Keywords:** differentially phosphorylated protein, okra, phosphoproteome, salt stress, TMT labeling

## Abstract

Soil salinization is a major environmental stresses that seriously threatens land use efficiency and crop yields worldwide. Although the overall response of plants to NaCl has been well studied, the contribution of protein phosphorylation to the detoxification and tolerance of NaCl in okra (*Abelmoschus esculentus* L.) seedlings is unclear. The molecular bases of okra seedlings’ responses to 300 mM NaCl stress are discussed in this study. Using a combination of affinity enrichment, tandem mass tag (TMT) labeling and high-performance liquid chromatography–tandem mass spectrometry analysis, a large-scale phosphoproteome analysis was performed in okra. A total of 4341 phosphorylation sites were identified on 2550 proteins, of which 3453 sites of 2268 proteins provided quantitative information. We found that 91 sites were upregulated and 307 sites were downregulated in the NaCl/control comparison group. Subsequently, we performed a systematic bioinformatics analysis including gene ontology annotation, domain annotation, subcellular localization, and Kyoto Encyclopedia of Genes and Genomes pathway annotation. The latter revealed that the differentially expressed proteins were most strongly associated with ‘photosynthesis antenna proteins’ and ‘RNA degradation’. These differentially expressed proteins probably play important roles in salt stress responses in okra. The results should help to increase our understanding of the molecular mechanisms of plant post-translational modifications in response to salt stress.

## 1. Introduction

Okra (*Abelmoschus esculentus* L.), an annual herb of Malvaceae family, is native to Africa and India [1,2]. It is not only a nutrient-rich vegetable that is used in traditional Chinese medicines, but also has a high culinary value. As a very important crop and vegetable, it is cultivated in many temperate and subtropical parts of the world [3]. Owing its high oil production rate and great ecological adaptability, okra is a potential bioenergy crop [4]. Most reports focus on its biological characteristics and cultivation techniques [5,6,7], chemical composition and medicinal value [8,9,10], and tissue culture [11,12]. However, there have been few studies on the salt tolerance of okra.

Salt stress is an important environmental condition that limits plant growth and decreases crop productivity. Irrigation water containing trace amounts of sodium chloride (NaCl) can increase soil salinity [13,14]. Once NaCl is absorbed by plant roots, the increased accumulation of salt in plants will cause ionic toxicity, hyperosmotic stress, and oxidative damage, damaging metabolic processes and reducing photosynthetic efficiency [14,15]. Plants have evolved various internal and external response strategies, resulting in the potential to adapt to salt stress by regulating ionic homeostasis, as well as increasing salt tolerance [16]. In *Arabidopsis thaliana*, the Salt Overly Sensitive 1 (SOS1)—a gene encoding a plasma membrane-localized Na^+^/H^+^ antiporter—maintains the intracellular Na^+^/K^+^ homeostasis [17]. The overexpression of *AtSOS1* enhances salt tolerance in transgenic Arabidopsis [18]. Calcium (Ca^2+^) signal transduction is a common signaling pathway that responds to the adverse environment encountered by eukaryotic cells [19]. Salt stress increases the Ca^2+^ concentration in the cytoplasm. A myristoylated Ca^2+^-binding protein—SOS3—has been proposed to sense this signal and physically interact with and activate a Ser/Thr protein kinase, SOS2 [20]. SOS1 is one of the downstream targets of SOS3–SOS2 complex. In addition, the phosphorylation of SOS1 by the SOS2–SOS3 complex could improve the salt tolerance of yeast [21]. Reactive oxygen species (ROS), as toxic by-product of normal cell metabolism, play a vital role in stress perception and signal transduction [22,23]. The ROS, particularly hydrogen peroxide (H_2_O_2_), accumulate under salt stress [24]. Oxidative stress-activated mitogen-activated protein triple-kinase 1 (OMTK1), a novel protein from alfalfa, is activated by H_2_O_2_ and functions in the activation of H_2_O_2_-induced cell death [25]. 

The transduction of extracellular signals often relies on protein post-translational modifications (PTMs) of proteins. Phosphorylation is the most researched and best understood PTM, and it can lead to changes in conformation, protein–protein interactions and protein activity [26]. In eukaryotic cells, protein phosphorylation occurs mainly at serine, threonine and tyrosine residues [27]. The technological development of phosphoproteomics provides a new opportunity for the wider identification of phosphorylation sites. The first large-scale phosphorylation proteome research method combines two-dimensional gel electrophoresis and mass spectrometry to identify spots [28]. Recently, isobaric tags for relative and absolute quantitation-based and tandem mass tags (TMTs)-based quantitative proteomics approaches were developed for large-scale protein quantification [29,30,31]. Large-scale scans of induced phosphoproteins have been performed in order to characterize plant responses to mechanical wounding [32], osmotic stress [33,34], drought [35], salinity [36], and high temperature [37]. Large numbers of phosphorylation proteomic analyses have been carried out in different plant species, such as Arabidopsis [38], rice (*Oryza sativa*) [39], maize (*Zea mays*) [15], bread wheat (*Triticum aestivum*) [35], and common bean (*Phaseolus vulgari*) [34]. These studies mainly focused on a few model plants, but our understanding of protein phosphorylation modifications underlying salt stress in okra is far from comprehensive. In this study, we investigated phosphorylation-modified proteins involved in the early stages of salt stress response in okra by employing TMT label-based quantitative proteomics.

## 2. Results

### 2.1. Quantitative Phosphoproteomic Data Analysis

**Figure 1:**
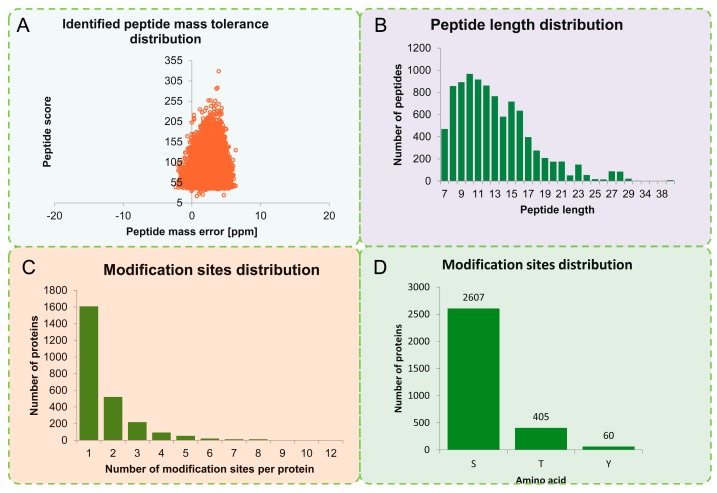
Quality control (QC) validation of mass spectrometer (MS) data. (**A**) Mass error distribution of all identified phosphorylated peptides. (**B**) Length distribution of all identified phosphorylated peptides. (**C**) Modification phosphorylated sites distribution of all identified peptides. (**D**) The distribution of phosphosites between serine (S), threonine (T), and tyrosine (Y) residues.

Through affinity enrichment and then using the LC-MS/MS approach, the phosphoproteomic changes in okra seedlings treated with salt stress were analyzed. The MS data validation is shown in Figure 1. The mass error of all the identified peptides was confirmed. The distribution of the mass error was near zero and most were less than 0.02 Da, indicating that the mass accuracy of the MS data fitted the requirements (Figure 1A). The lengths of most peptides were between eight and 20 amino acids (aa), which agreed with the tryptic peptides’ properties (Figure 1B). 

The pair-wise Pearson’s correlation coefficients among the six samples showed sufficient reproducibility (Figure S1). The detailed information of identified peptides, including peptide sequences, matching scores, precursor charges, modifications and delta masses, can be found in Table S1.

### 2.2. Annotation and Classification of all Identified Phosphorylated Proteins in Okra

A total of 4341 phosphorylation sites were identified on 2550 proteins, of which 3453 sites of 2268 proteins provided quantitative information (Table S2). Approximately 63.05% of the peptides were modified at a single site, 20.42% were altered at two sites, and 8.54% at three sites (Figure 1C). To ensure that the results were highly reliable, we filtered the identification data using a criterion of a localization probability >0.75 and ultimately identified 3072 phosphorylation sites on 2175 proteins, of which 2241 sites of 2027 proteins provided quantitative information. The filtered data were used for subsequent bioinformatics analyses. Among the phosphorylation sites, 2607 (84.86%) involved a serine residue, 405 (13.18%) a threonine, and 60 (1.95%) a tyrosine (Figure 1D).

**Figure 2:**
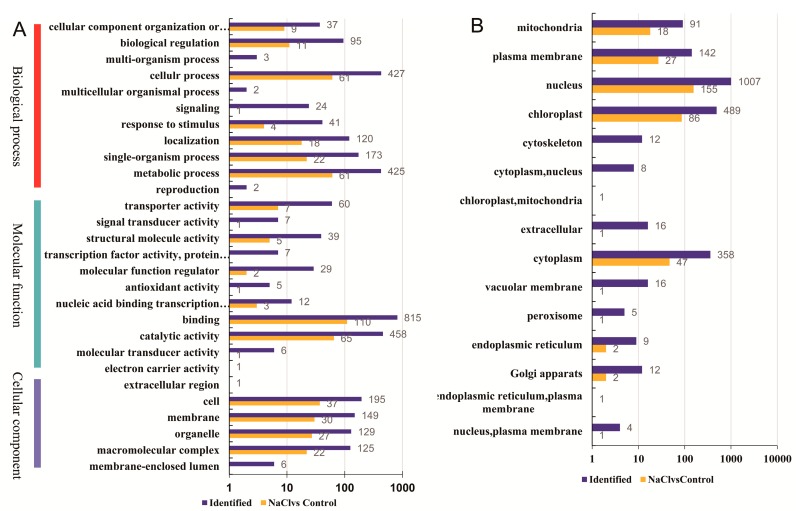
Classification of all identified phosphorylated proteins and differentially phosphorylated proteins (DPPs). (**A**) Gene Ontology (GO) analysis of all identified phosphorylated proteins and DPPs. All proteins were classified by GO terms based on three categories: molecular function, biological process and cellular component. (**B**) Subcellular classify of phosphorylated proteins and DPPs.

To understand their functions, all identified phosphorylated proteins were annotated by GO terms based on the three major categories, namely biological process, cellular component and molecular function (Figure 2A). For the biological process category, ‘cellular process’ (427 proteins), ‘metabolic process’ (425 proteins), and ‘single-organism process’ (173 proteins) were the top dominant terms; for the molecular function category, the dominant terms were ‘binding’ (815 proteins), ‘catalytic activity’ (458 proteins), and ‘transporter activity’ (60 proteins); and for the cellular component category, most proteins were related to ‘cell’ (195 proteins), ‘membrane’ (149 proteins), and ‘organelle’ (129 proteins). Most identified proteins were grouped into 15 subcellular component categories predicted by Wolfpsort software, including nucleus (1007 proteins), chloroplast (489 proteins), and cytoplasm (358 proteins) (Figure 2B). The detailed information, including protein IDs, GO, KEGG, and domain categories, 238 subcellular localizations and functional enrichments of all identified proteins, are listed in Table S3.

### 2.3. Peptide Motifs Associated with Phosphorylation

A total of 2514 sequences containing the 13 residues, with six upstream and six downstream residues surrounding each of the phosphorylation sites, were obtained (Figure S2). Of these, 2255 (89.70%) were centered on a serine residue, 237 (9.43%) on a threonine, and 22 (0.87%) on a tyrosine. The serine phosphorylation category included 29 overpresented motifs: the most frequent motifs were ‘sP’ (355 occurrences) and ‘PxsP’ (182 occurrences), followed by ‘Rxxs’ (151 occurrences) and ‘RSxs’(116 occurrences). ‘tP’ was the most frequent motif in the threonine phosphorylation category.

### 2.4. Differentially Phosphorylated Proteins in Response to NaCl Treatment

**Figure 3:**
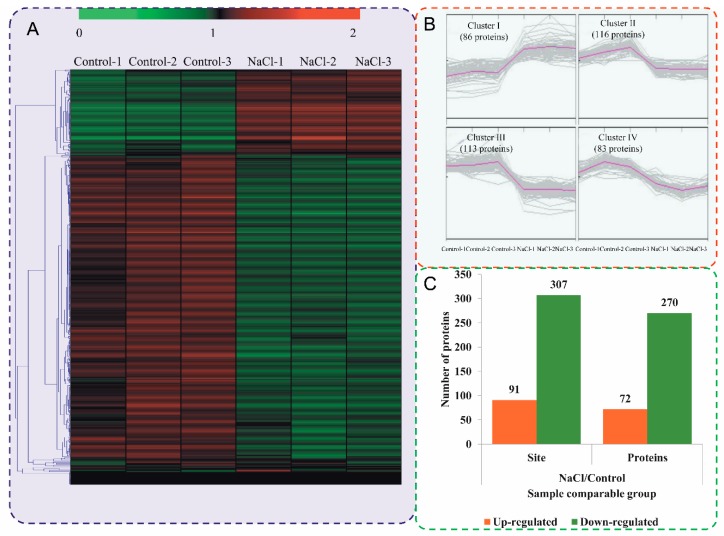
Impacts of salt stress treatment on phosphorylation proteome levels in okra. (**A**) Expression profiles of the DPPs in response to salt stress. (**B**) All DPPs were analyzed and clustered into four major Clusters by K-means method. (**C**) The numbers of up- and downregulated phosphorylated proteins and phosphorylated sites in the salt treatment seedlings compared to the control seedlings.

To compare the differentially phosphorylated proteins (DPPs) between different samples, expression profiles of the proteins are shown in a heatmap (Figure 3A). To reflect the changing trends among the six samples, all of the DPPs were categorized into four clusters with MeV software using the K-means method. The proteins in cluster I showed high accumulations in the NaCl stress-treated samples, while the rest of the proteins were downregulated in the NaCl stress-treated samples (Figure 3B). Among the DPPs, 72 proteins were upregulated and 270 proteins were downregulated at 48 h after NaCl treatment compared with the control (Figure 3C). All DPPs were classified into 11 subcellular components according to their subcellular localizations (Figure 2B), including 155 nuclear-localized DPPs, 86 chloroplast-localized DPPs, and 47 cytoplasm-localized DPPs. The detail information of DPPs and differential phosphosites are listed in Table S4.

**Figure 4:**
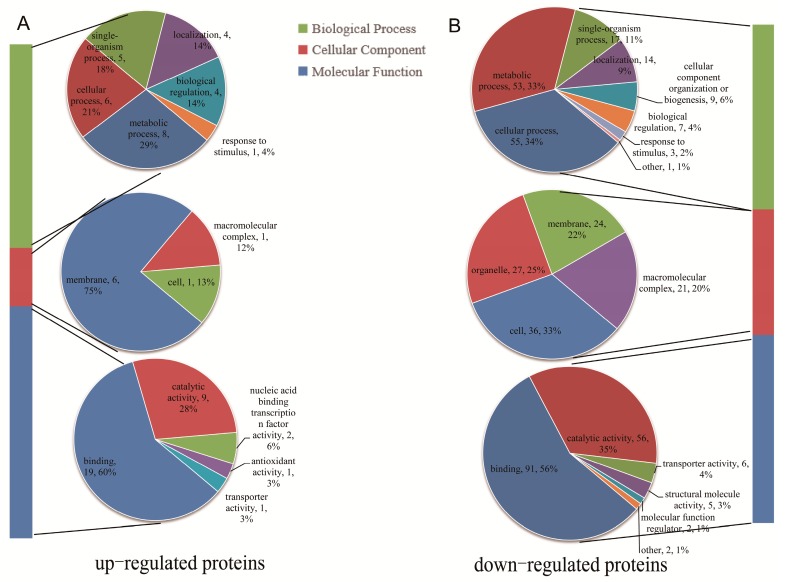
GO annotation analysis of DPPs. Distribution of the upregulated (**A**) and downregulated (**B**) proteins with GO annotation. Different color blocks represented different terms, including cellular component, molecular function, and biological process. Number of the upregulated proteins in each second level term was showed in a pie chart.

To predict the biological functions, all DPPs were assigned to GO terms. For upregulated proteins, the largest numbers of DPPs were found in ‘metabolic process’ (eight proteins) in the biological process GO term, in cellular component, the most frequent was ‘membrane’ (six proteins), while, in molecular function, ‘binding’ (19 proteins) was the most highly represented group (Figure 4A). For downregulated proteins, the most enriched biological process GO term was ‘cellular process’; within “cellular component”, the most enriched was related to ‘cell’ (36 proteins); and the most enriched term within molecular function was ‘binding’ (91 proteins) (Figure 4B). In total, 41 DPPs were involved in membrane and transport proteins according to the GO terms and their subcellular localizations (Table 1). Eight of them were induced by the NaCl treatment. The GO enrichment analysis revealed that the molecular function GO term of upregulated DPPs most significantly enriched was ‘oxidoreductase activity, acting on NAD(P)H’ and that the biological process’ GO term most significantly enriched was ‘metal ion transport’ (Figure S3). For the downregulated DPPs, the molecular function GO terms most significantly enriched was ‘transferase activity, transferring glycosyl groups’ while the biological process GO term most significantly enriched was ‘DNA metabolic process’ (Figure S3).

### 2.5. Enrichment Analysis of the DPPs under the NaCl Treatment 

**Figure 5:**
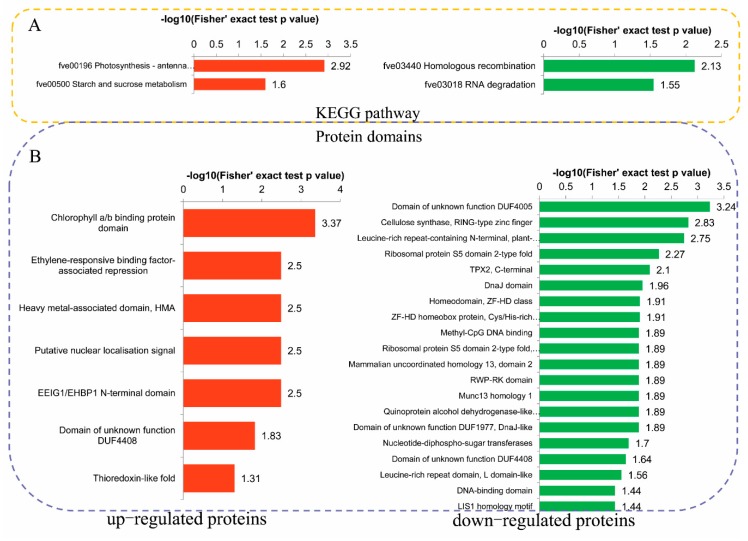
Kyoto Encyclopedia of Genes and Genomes (KEGG) and domain enrichment analysis of the DPPs in okra after salt stress treatment. (**A**) Significantly enriched KEGG pathways of the DPPs. (**B**) Significantly enriched protein domains of the DPPs.

**Figure 6:**
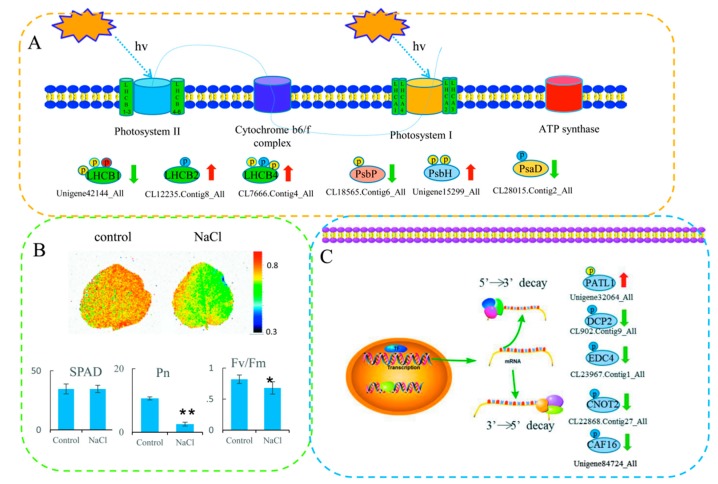
DPPs involved in photosynthesis and RNA degradation. (**A**) DPPs involved in photosynthesis. (**B**) The value of Fv/Fm, SPAD, Net photosynthetic rate (Pn) compared to respective controls in okra leaves after NaCl treatments. (**C**) DPPs involved in RNA degradation. The phosphorylated serine (S), threonine (T), and tyrosine (Y) residues are shown in blue, yellow, and red, respectively. LHCB1/2/4: light harvesting complex II chlorophyll a/b binding protein 1/2/4; PsbP: photosystem II oxygen-evolving enhancer protein 2; PsbH: photosystem II protein H; PsaD: PSI reaction center subunit II; PATL1: DNA topoisomerase 2-associated protein PAT1; DCP2: mRNA-decapping enzyme subunit 2; EDC4; enhancer of mRNA-decapping protein 4; CNOT2: CCR4-NOT transcription complex subunit 2; CAF16: CCR4-NOT complex subunit 16.

The KEGG enrichment analysis revealed that the upregulated DPPs were most strongly associated with ‘Photosynthesis—antenna proteins’ and ‘Starch and sucrose metabolism’ pathways. The downregulated DPPs were most strongly associated with ‘Homologous recombination’ and ‘RNA degradation’ pathways (Figure 5A). We further summarized the DPPs involved in photosynthesis and RNA degradation (Figure 6). In total, six proteins related to photosynthesis were identified, including light-harvesting complex II (LHCII) chlorophyll a/b-binding protein 1/2/4, photosystem II oxygen-evolving enhancer protein 2, photosystem II protein H (PsbH), and PSI reaction center subunit II. In total, 11 phosphorylated sites were identified in these proteins (Figure 6A). We also measured some physiological parameters owing to differences in some proteins in the photosynthetic pathways. The Fv/Fm image is shown in Figure 6B. The maximum quantum efficiency of photosystem II (PSII) (Fv/Fm) and Pn values decreased significantly in NaCl treatments compared with their respective controls, but the SPAD values did not change significantly. Five proteins related to RNA degradation were identified, including DNA topoisomerase 2-associated protein PAT1, mRNA-decapping enzyme subunit 2 (DCP), enhancer of mRNA-decapping protein 4, CCR4-NOT transcription complex subunit 2, and CCR4-NOT complex subunit 16 (Figure 6C). 

A protein domain enrichment analysis revealed that the domains ‘Chlorophyll a/b binding protein domain’, ‘Ethylene-responsive binding factor-associated repression’, and ‘Heavy metal-associated domain (HMA)’, were enriched in the upregulated DPPs, while the downregulated DPPs were most strongly associated with ‘Domain of unknown function DUF4005’, ‘Cellulose synthase, RING-type zinc finger’, and ‘Leucine-rich repeat-containing N-terminal, plant-type’ (Figure 5B). 

### 2.6. Protein–Protein Interactions (PPIs) of DPPs

**Figure 7:**
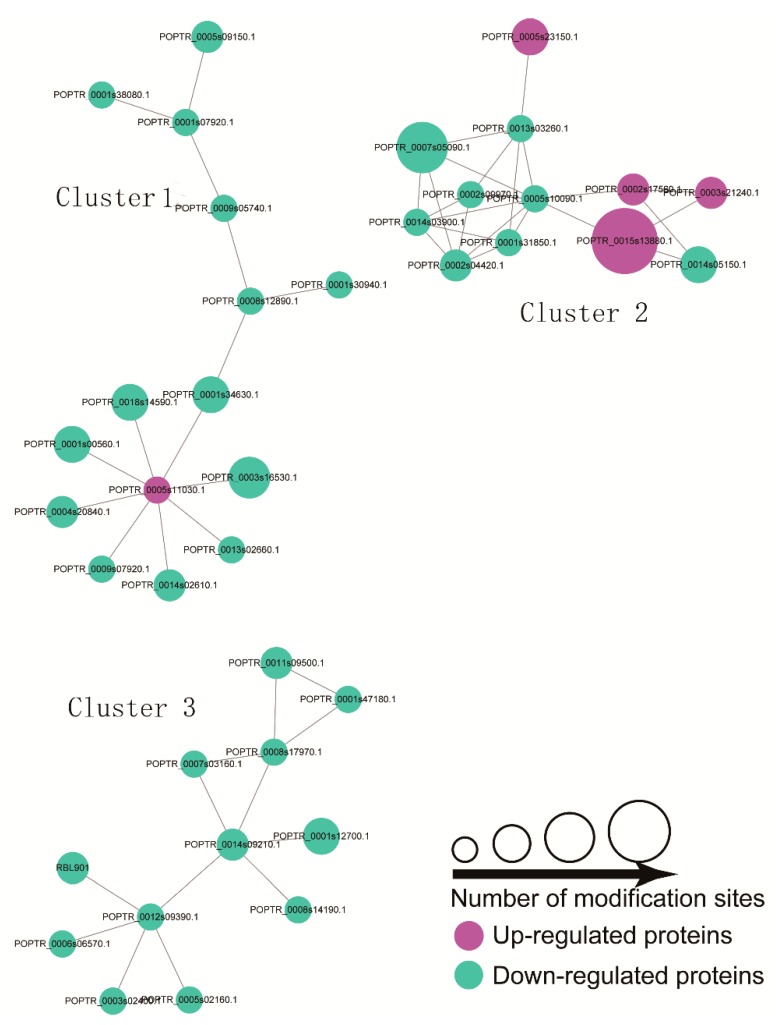
Interaction network of DPPs analyzed by Cytoscape sofware (version 3.0.1). The upregulated and downregulated proteins in the clusters are shown in cyan and green, respectively. Detailed information on node and proteins can be found in Tables S5 and S6.

A PPI network analysis can reveal the relationship between biological functions of different phosphorylated proteins. The high quality PPI maps are shown in Figure 7, and the detailed node and network information are listed in Tables S5 and S6. A total of 39 DPPs (76 modified sites), including five up- and 34 downregulated peptides, was displayed in the PPI network (Figure 7). Three enriched interaction clusters were identified from our analysis. For cluster 1, six ‘phosphorylation’-related proteins (downregulated), three ‘binding’-related proteins (downregulated), a small subunit ribosomal protein S6e (downregulated), a protein-tyrosine phosphatase-like protein, a WD40-repeat-containing domain protein (downregulated), and a PPM-type phosphatase domain (upregulated), have been included. Four ‘Ribosome’-related proteins (downregulated) and three ‘Spliceosome’-related proteins (upregulated) were identified in cluster 2. For cluster 3, six nucleus-localized proteins, three mitochondria-localized proteins, two chloroplast-localized proteins, and a chloroplast-localized protein cytoplasm have been identified. All DPPs in cluster three were downregulated.

## 3. Discussion

Plant responses to salt stress are mediated by complex molecular mechanisms, including signal transduction, and the transcription and translation of salt stress-related genes. Reversible protein phosphorylation is a key regulatory mechanism operating in response to abiotic and biotic stresses in plant [40,41]. In this study, a TMT-based quantitative phosphoproteomic approach was used to study the response of okra shoots to salt stress (Figure 1). Many phosphorylation sites were identified, and some phosphorylated proteins that might be involved in salt stress response were identified (Figure 3). These salt-responsive phosphoproteins may play important roles in salt stress signaling and response in okra shoots.

The first method for large-scale phosphorylation proteomics involved two-dimensional gel electrophoresis, with spots identified by mass spectrometry [28]. However, it is difficult to identify proteins of low abundance, low molecular weight (<15 kDa) or high molecular weight (>150 kDa); superacidic or basic proteins; or hydrophobic proteins by this method [42]. Recently, an MS/MS-based analysis strategy using isobaric tag for relative and absolute quantitation or TMT-labeling was developed for large-scale phosphorylated protein quantification [43,44]. In our study, 4341 phosphorylation sites, 2550 phosphorylated proteins, and 342 DPPs were identified (Table S2). The large number of identified phosphorylated proteins gave us the opportunity to conduct a more in-depth and comprehensive analysis of salt-responsive proteins. ‘sP’ and ‘Rxxs’ are the two most frequently occurring motifs for phosphoserine, and this was confirmed in okra. The ‘sP’ motif, which provides a target for cAMP- and cGMP-dependent protein kinase C, CDK protein kinase 2, Ca-dependent protein kinases, mitogen-activated protein kinase, receptor-like kinase, sucrose nonfermenting 1-related protein kinase 2 and STE20-like kinase, has also been identified as being overrepresented in other plants [45] (Figure S2). Meanwhile, the ‘Rxxs’ motif has been reported to be associated with mitogen-activated protein kinase kinase, protein kinase A, and calmodulin-dependent protein kinase [35,45]. 

In the face of various biotic and abiotic stresses, plants gradually regulate water and ion transport mechanisms [46]. Maintaining cell osmotic potential under salt stress is a major challenge for plant growth and development [47]. In this study, several membrane proteins and ion transporters were shown to be differentially phosphorylated under salt stress, suggesting their phosphorylation of these proteins may regulate osmotic balance in okra under salt stress (Table 1). Under salt stress, several transporters of sugars, amino acids or ions were differentially phosphorylated in okra, and some might be involved in osmotic regulation. Three phosphorylation sites of two multidrug/pheromone exporter proteins in the ATP-binding cassette (ABC) transporter subfamily B decreased, and a white-brown complex ABC transporter protein was induced after the salt treatment in this study. ABC transporters are a class of transmembrane proteins that are ubiquitous in prokaryotes and eukaryotes [48]. Most ABC transporters bind to adenosine triphosphate (ATP) and release energy by hydrolyzing ATP to regulate the transmembrane transport of substances [48]. Plant ABC transporters play key roles in plant secondary metabolite transport and accumulation, phytohormone transport, lipid metabolism, exogenous toxin detoxification, and plant disease resistance [49]. Some transporters and iron pumps, such as an amino acid transporter (Unigene80449_All), a sugar phosphate transporter (Unigene66780_All), an oligopeptide transporter (Unigene78857_All), mechanosensitive ion channel protein 8-like (CL18.Contig22_All), phosphate transporter PHO1 homolog 3(CL27908.Contig8_All), a sugar transporter (CL2495.Contig20_All), UDP-glucuronic acid/UDP-*N*-acetylgalactosamine transporter (CL7033.Contig3_All), and vacuolar cation/proton exchanger 1a (CL27084.Contig3_All) were repressed by salt stress. Changes in phosphorylation status of these transporters after salt stress treatment suggest that they may play a role in regulating ion and small molecule concentrations to balance cell osmotic potential and reduce cytoplasmic water loss. Four phosphorylation sites of three cellulose synthase proteins decreased after salt treatment in this study. The cell wall plays an important role in maintaining cell morphology, and the cellulose synthase family proteins is involved in cell wall formation was regulated at phosphorylation level [50,51].

We detected changes in some phosphorylated proteins involved in photosynthesis after the salt treatment (Figure 6A). Similar results were also reported found in *A. thaliana* [24]. Phosphorylation at sites on three light-harvesting II complex (LHCII) proteins—LHB1B2, LHCb4.2, and LHCb1.2—were induced following exposure to salt or H_2_O_2_ stresses, as were sites on PsbH and PsaP [24]. The phosphorylation of LHCII proteins adapts to environmental changes [52]. The phosphorylation of LHCII participates in the energy distribution between the two photosystem, and the signal transduction between light reception and the phosphorylation of LHCII is correlated with the redox state of the proton quinone pool. Thus, a reduction of proton quinone leads to the activation of the kinase [53]. Phosphorylation of PsbH proteins regulates the whole membrane network rearrangement of plant thylakoids [24]. Therefore, the phosphorylation of chloroplast proteins affects the redox state [54]. Several DDPs involved in the mRNA degradation pathway (Figure 6C). Removing the 5′ caps of mRNA is an important step in post-transcriptional regulation, because it represents a movement away from active translation and is a prerequisite for the rate-limiting degradation of exogenous ribonucleases [55]. The correlation between transcriptional level and protein abundance was poor after the salt treatment [56]. The transcriptional level was mainly disconnected from the active polymer during dehydration stress [57], indicating that the post-transcriptional regulation mechanism was activated in the early dehydration reaction [58,59]. Our data identified that phosphorylation of DNA topoisomerase 2-associated protein PAT1 increased at the threonine residue, while mRNA-decapping enzyme subunit 2, enhancer of mRNA-decapping protein 4, CCR4-NOT transcription complex subunit 2, and CCR4-NOT complex subunit 16 decreased at serine residues following the NaCl treatment, which suggests that mRNA decapping is altered in response to salt stress.

Ca^2+^ is involved in plant signal transduction under environmental stress, and CDPks play a key role in Ca^2+^ mediated signal transduction pathway [51]. A calmodulin-like protein(CL9944.Contig4_All) and CDPk protein (Unigene71267_Alll) were decreased at phosphorylation level under NaCl treatments, indicating the Ca^2+^-mediated pathway participates in salt stress signaling in okra shoots (Figure 7 and Table S5). Protein degradation in plants may be activated under salt stress. Previous proteomic studies have identified a large number of salt stress-related proteins related to protein synthesis, folding and degradation [47,60,61]. In the present study, we observed that five ribosomal protein (40S ribosomal protein S6A, CL2686.Contig12_All; ribosomal protein S15e, CL9991.Contig2_All; ribosomal protein S9, CL19354.Contig5_All; ribosomal protein S6e, Unigene79731_All; ribosomal protein S6-like, Unigene55159_All) decreased at the phosphorylation level under NaCl treatment (Figure 7 and Table S4). The reduction of these phosphoribosomal proteins supports the view that salt stress usually inhibits protein synthesis [36]. Ribosomal protein S6 (rps6), a component of the 40S ribosomal subunit, whose phosphorylation status can be affected by osmotic stress via TOR pathway [62,63].

## 4. Materials and Methods 

### 4.1. Plant Materials and Treatment

Seeds of the okra cultivar ‘Wufu’ were used in this study. The seeds were disinfected with 1% sodium hypochlorite for 10 min and washed with distilled water three times. Seeds were sown in plastic trays of turfy soil. One week after germination, half-strength Hoagland’s nutrient solution was used for irrigation every 3 days. Seedlings were grown in an artificial illumination incubator with a photoperiod of 12-h light/12-h dark, relative humidity of 60%, and light intensity of 300 μmol m^−2^ s^−1^. Three weeks after germination, 20 mL of 300 mmol L^−1^ NaCl was applied. After 48 h treatment, the chlorophyll fluorescence images were captured using a FluorCam 800 imaging system (PhotonSystems Instruments, Brno, Czechia Republic) as previously described by Feng et al. [64]. After acquiring the chlorophyll fluorescence imaging data, the aboveground parts of the okra plants were harvested to measure the chlorophyll content using a SPAD meter (502DL Plus, SPECTRUM, Illinois, IL, USA). Net photosynthesis (Pn) was measured by using a portable photosynthesis system (LI 6400, LI-COR, Lincoln, NE, USA) with a red/blue LED light source at 1000 µmol m^−2^ s^−1^ Photosynthetic Photon Flux Density.

### 4.2. Protein Extraction and Digestion

At 48 h after salt treatment, the aboveground seedling samples were fully ground to a powder in liquid nitrogen. Four-fold volume phenol extraction buffer (containing 10 mM dithiothreitol, 1% protease inhibitor, and 1% phosphatase inhibitor) was added to the samples of each group, and ultrasonic pyrolysis was performed in high intensity ultrasonic processor (Scientz, Ningbo, China). An equal volume of Tris-saturated phenol was added and centrifuged (4 °C, 10 min, 5000 *g*). The supernatant was removed and precipitated overnight with a 5× volume of 0.1 M ammonium acetate/methanol solution. The precipitated protein was washed successively with methanol and acetone. The protein was redissolved in 8 M urea, and the protein concentration was determined using a bicinchoninic acid assay (BCA) kit (CW0014; CWBIO, Beijing, China), according to the manufacturer’s instructions.

For trypsin digestion, dithiothreitol was added to the protein solution, resulting in a 5-mM final concentration, and reduced for 30 min at 56 °C. Then, iodoacetamide was added to an 11-mM final concentration and the samples were incubated at room temperature for 15 min in the dark. Finally, the urea concentration of the samples was diluted to below 2 M by adding 100 mM triethyl ammonium bicarbonate. Trypsin was added at 1:50 mass ratio (trypsin: protein) for the first hydrolysis overnight at 37 °C and at 1:100 (trypsin: protein) mass ratio for a second 4-h digestion.

### 4.3. TMT Labeling and High-Performance Liquid Chromatography (HPLC) Fractionation 

After trypsin digestion, peptides were desalted using Strata X C18 solid-phase extraction (Phenomenex, Torrance, CA, USA) and vacuum freeze-dried. The peptides were dissolved in 0.5 M triethyl ammonium bicarbonate and labeled using a TMT kit (ThermoFisher, Shanghai, China) according to the manufacturer’s instructions. Briefly, the labeled reagent was dissolved in acetonitrile after thawing and incubated at room temperature for 2 h after mixing with the peptides. Then, the peptide mixture was desalted and freeze-dried under vacuum.

The peptides were fractionated by high pH reversed HPLC with a Thermo Betasil C18 column (5 μm diameter, 10 mm inner diameter, 250 mm length). The operation was as follows: peptides were first separated into 60 fractions using a gradient of 8% to 32% acetonitrile (pH 9.0) over 60 min. Then, the peptides were pooled into 18 fractions and dried by vacuum centrifugation.

### 4.4. Affinity Enrichment

The peptides were dissolved in a concentrated buffer solution (50% acetonitrile/6% trifluoroacetic acid), and the supernatant was transferred to the immobilized metal ion/metal chelate material, which had been washed in advance. The peptide–resin mixture were placed on a rotary table and gently shaken. After this incubation period, the resin was washed three times successively with buffer solutions of 50% acetonitrile/6% trifluoroacetic acid and 30% acetonitrile/0.1% trifluoroacetic acid. The phosphopeptides were then eluted with 10% ammonia solution. The eluent was collected and vacuum freeze-dried. After being drained, the eluent was de-salted using C18 ZipTips according to the manufacturer’s instructions. After vacuum freeze-drying, the eluent was used for liquid chromatography–tandem mass spectrometry (LC-MS/MS) analysis.

### 4.5. LC-MS/MS Analysis

The peptides were dissolved in an aqueous solution of 0.1% (*v*/*v*) formic acid and separated using an EASY-nLC 1200 ultrahigh-performance liquid phase system (Thermo Fisher Scientific, Waltham, MA, USA). The liquid gradient settings were as follows: 0–38 min, 4%–22% B; 38–52 min, 22%–32% B; 52–56 min, 32%–80% B. Solvent B was an aqueous solution containing 0.1% formic acid and 90% acetonitrile. The flow rate was maintained at 450 nL/min.

The peptides were ionized using an NSI ion source and then analyzed by Q Exactive^TM^ HF-X mass spectrometry (ThermoFinnigan, Somerset, NJ, USA). The ion source voltage was set at 2.0 kV, and the peptide parent ions and their secondary fragments were detected and analyzed using a high-resolution Orbitrap. The scanning range of the first-order mass spectrometry was set to 350–1600 m/z, and the scanning resolution was set to 60,000. The fixed starting point of the second-order mass spectrum scanning range was 100 m/z, and the second-order scanning resolution was set at 30,000. In the data acquisition mode, the data-dependent scanning program was used, in which, the parent ions of the first 20 peptides with the highest signal intensity were selected to enter the higher-energy C-trap dissociation collision pool successively after the first scan. The fragmentation energy was set at 28%, and the second-order mass spectrometry analysis was performed successively. To improve the effective utilization rate of mass spectrometry, the automatic gain control was set to 1E5, the signal threshold was set to 5E3, the maximum injection time was set to 100 ms, and the dynamic exclusion time of the tandem mass spectrometry scanning was set to 15 s to avoid the repeated scanning of parent ions.

### 4.6. Database Search

The resulting MS/MS data were queried against our previously published okra transcriptome data (NCBI Sequence Read Archive database accession: SRP130180) using the Maxquant search engine (v.1.5.2.8) concatenated with the reverse decoy database [65]. The enzyme digestion mode was set as Trypsin/P. The number of missing cut points was set as 2. The minimum length of the peptide segment was set to seven amino acid (aa) residues. The maximum modification number of modifications for a peptide segment was set as five. First search and Main search were set as 20 PPM and 5 PPM, respectively, and the mass error tolerance of secondary debris ions was 0.02 Da. Cysteine alkylation was set as the fixed modification, and the alterable modification was methionine oxidation. The quantitative method was set as tmt-6plex, and the FDR for protein identification and PSM identification were set at 1%.

### 4.7. Annotation Methods and Functional Enrichment

The gene ontology (GO) annotation of the proteome was derived from the UniProt-GOA database (http://www.ebi.ac.uk/GOA/). First, the identified protein IDs were converted to UniProt IDs and then mapped to GO IDs. The InterProScan software was used to annotate the protein’s GO function based on the protein sequence alignment method if the proteins were not annotated by the UniProt-GOA database. For each category, a two-tailed Fisher’s exact test was employed to test the enrichment of the differentially expressed protein (DEP) against all identified proteins. A GO annotation with a corrected *p*-value < 0.05 was considered significant. The Kyoto Encyclopedia of Genes and Genomes (KEGG) database was used to annotate protein pathways. The InterPro domain database (http://www.ebi.ac.uk/interpro/) was used to analyze identified the functional descriptions of protein domains. A pathway with a corrected *p*-value < 0.05 was considered significant. Wolfpsort (http://www.genscript.com/psort/wolf_psort.html), a subcellular localization predication software, PSORT/PSORT II version, was used to predict subcellular localization. The InterPro database was searched, and a two-tailed Fisher’s exact test was employed to test the enrichment of the DEP against all identified proteins. Protein domains with a *p*-value < 0.05 were considered significant. The software motif-x was used to analyze the models of sequences that contained the amino acids in specific positions of modify-13-mers (six amino acids upstream and downstream of the site) in all protein sequences. Additionally, all the database protein sequences were used as background database parameters, and the other parameters were set at default.

### 4.8. Protein–Protein Interaction Network

All differentially phosphorylated proteins that were classified as identifiers were queried against the STRING database version 10.5 (http://string-db.org/cgi/input.pl) for protein–protein interactions. The differential protein interactions were extracted according to the confidence score of >0.7 (high confidence). Then, the R package “network workd3” tool (https://cran.r-project.org/web/packages/networkD3/) was used to visualize the differential protein interaction networks.

## 5. Conclusions

In summary, we presented a comprehensive large-scale phosphoproteome of okra using a combination of affinity enrichment and TMT labeling high-resolution LC-MS/MS analysis. A total of 4341 phosphorylation sites were identified on 2550 proteins. We also investigated the DPPs that changed after the exposure of okra seedlings to salt or water for 48 h. In total, 342 DPPs (399 phosphorylated sites) were identified, 72 (91 sites) of which were upregulated and 270 (307 sites) were downregulated under salt stress conditions. A number of DPPs were involved in photosynthesis and mRNA degradation pathway. Our data will provide a basic resource that will assist understanding of the molecular mechanisms involved in the responses of okra plants to salt stress.

## Figures and Tables

**Table 1:** Membrane and transport-related proteins differentially expressed under salt stress in okra leaves.

Protein Accession	Ratio	Protein Description	Modified Sequence
Unigene80449_All	0.693	amino acid transporter	TIGDIT(ph)PK
CL5416.Contig1_All	0.692	ATP binding protein, putative	AS(ph)AFVLGK
CL7525.Contig9_All	0.745	Auxin-induced protein 5NG4, putative	AKEENLGSS(ph)NQK
Unigene36424_All	1.664	Bidirectional sugar transporter SWEET15	VVEDTKVPEETNNS(ph)IAGLSK
CL5012.Contig7_All	1.375	calcium-transporting ATPase 12, plasma membrane-type-like	FLSSS(ph)FAPK
Unigene92546_All	0.75	cellulose synthase catalytic subunit	LSMAS(ph)PGPVGGK
Unigene92535_All	0.727	CESA6	TVS(ph)GEIATPDNQFVR
Unigene92535_All	0.677	CESA6	HDS(ph)DS(ph)GPKPLK
Unigene92544_All	0.644	CESA6	TTS(ph)GPLGPSEK
Unigene66780_All	0.684	gb|EEE94510.1| predicted: Sugar phosphate transporter protein	SLS(ph)LPTTR
Unigene78857_All	0.731	gb|EEF37920.1| oligopeptide transporter, putative	QAPETEIS(ph)IKK
Unigene84207_All	0.726	gb|EEF44433.1| transferase, transferring glycosyl groups	TSS(ph)SLGLLFK
CL8578.Contig9_All	0.557	golgi SNAP receptor complex member 1	S(ph)LFGDVQGK
CL4324.Contig7_All	0.73	golgi snare 11 protein, putative	S(ph)TFGGINSK
Unigene94022_All	1.308	hypothetical protein ARALYDRAFT_893642	LKT((ph))LELQNK
CL18.Contig22_All	1.33	mechanosensitive ion channel protein 8-like	GVS(ph)FDNSPLR
CL2667.Contig3_All	0.756	multidrug/pheromone exporter, MDR family, ABC transporter family	NLSYSYS(ph)TGADGR
CL4242.Contig2_All	0.623	multidrug/pheromone exporter, MDR family, ABC transporter family	NSVSS(ph)PIITR
CL2667.Contig3_All	0.72	multidrug/pheromone exporter, MDR family, ABC transporter family	LSHSLS(ph)TK
CL2258.Contig6_All	0.638	Nucleoporin NUP53, putative	DFS(ph)IPAK
CL23428.Contig3_All	0.675	Patellin-3, putative	LKT(ph)MPTSD
CL27908.Contig8_All	0.656	Phosphate transporter PHO1 homolog 3	AFS(ph)GLTHR
Unigene2678_All	0.725	PREDICTED: protein transport protein Sec61 subunit β	GSAAAAAS(ph)LR
Unigene67918_All	1.344	protein binding protein, Zinc finger, putative	RMDS(ph)FFR
Unigene67918_All	1.475	protein binding protein, Zinc finger, putative	VIPST(ph)PR
CL2662.Contig7_All	1.41	Protein kinase domain	QPATSFMS(ph)AYK
CL2495.Contig20_All	0.639	sugar transporter, putative	HGS(ph)LVSQR
Unigene20607_All	0.753	transferase, transferring glycosyl groups, putative	RHSES(ph)GLEILNK
CL7033.Contig3_All	0.707	UDP-glucuronic acid/UDP-*N*-acetylgalactosamine transporter, putative	LS(ph)DGGGVPK
CL27084.Contig3_All	0.541	Vacuolar cation/proton exchanger 1a, putative	S(ph)DSLLVSK
CL530.Contig6_All	1.373	white-brown-complex ABC transporter family	MMIGAS((ph))PK
CL27656.Contig6_All	0.733	xylosyltransferase 1-like	TYYS(ph)QFR
Unigene52837_All	0.762	emb|CBI29277.3| PREDICTED: uncharacterized protein LOC100259097	S(ph)SFPSSSSSR
CL20053.Contig2_All	1.63	conserved hypothetical protein	GKS(ph)LPKPYIDR
CL2142.Contig22_All	0.718	gb|EEF33488.1| transporter, putative	GFSHVES((ph))K
CL23414.Contig54_All	0.727	PREDICTED: uncharacterized protein LOC100248394	TATHSTPVASHTDS((ph))FDSDSR
CL24219.Contig9_All	0.674	conserved hypothetical protein	KSS(ph)GPQSGGVTSSGR
CL2564.Contig3_All	0.734	hypothetical protein PRUPE_ppa000754mg	ETVPQGEYS(ph)LSHTSAPFR
CL27706.Contig6_All	0.65	hypothetical protein PRUPE_ppa002944mg	LAVS(ph)PGKVEGHR
CL7523.Contig8_All	0.64	gb|EEE91340.1| predicted protein	VSPEFSHPQSS(ph)SPMAK
CL7658.Contig6_All	0.676	gb|EEF00758.1| predicted protein	VASS(ph)PMKR
CL8864.Contig7_All	0.749	unknown protein	INS(ph)SPIVSR
Unigene68387_All	0.733	|gb|EEF48730.1|conserved hypothetical protein	KVS(ph)GPLESMGS(ph)MK
Unigene68390_All	0.705	|gb|EEF48730.1| conserved hypothetical protein	KS((ph))GELGK
Unigene68390_All	0.728	gb|EEF48730.1| conserved hypothetical protein	KVS((ph))GPLDSMGS(ph)MK
Unigene68390_All	0.638	gb|EEF48730.1| conserved hypothetical protein	KSNS(ph)GPLNR

## Supplementary Materials

Supplementary materials can be found at https://www.mdpi.com/1422-0067/20/6/1262/s1.

## Abbreviations

LC-MS	Liquid chromatography electrospray ionization tandem mass spectrometry
TMT	Tandem Mass Tag
KEGG	Kyoto Encyclopedia of Genes and Genomes
GO	Gene Ontology
SOS	Salt Overly Sensitive
ROS	Reactive oxygen species
PTM	post-translational modification
QC	Quality control
DPP	Differentially phosphorylated protein
LHCII	light-harvesting complex II
PsbP	photosystem II oxygen-evolving enhancer protein 2
PsbH	photosystem II protein H
PsaD	PSI reaction center subunit II
PATL1	DNA topoisomerase 2-associated protein PAT1
DCP2	mRNA-decapping enzyme subunit 2
EDC4	enhancer of mRNA-decapping protein 4
CNOT2	CCR4-NOT transcription complex subunit 2
CAF16	CCR4-NOT complex subunit 16
PPI	Protein–Protein Interaction

## References

[1] Kumar S., Dagnoko S., Haougui A., Ratnadass A., Pasternak D., Kouame C. (2010). Okra (*abelmoschus* spp.) in west and central africa: Potential and progress on its improvement. Afr. J. Agric. Res..

[2] Molfetta I., Ceccarini L., Macchia M., Flamini G., Cioni P.L. (2013). *Abelmoschus esculentus* (L.) moench. And abelmoschus moschatus medik: Seeds production and analysis of the volatile compounds. Food Chem..

[3] Karakoltsidis P.A., Constantinides S.M. (1975). Okra seeds. New protein source. J. Agric. Food Chem..

[4] Moosavi S.A., Aghaalikhani M., Ghobadian B., Fayyazi E. (2018). Okra: A potential future bioenergy crop in Iran. Renew. Sustain. Energy Rev..

[5] Martin F.W., Rhodes A.M. (1983). Seed characteristics of okra and related abelmoschus species. Qual. Plant.

[6] Düzyaman E. (1997). Okra: botany and horticulture. Horticultural Reviews.

[7] Bisht I.S., Patel D.P., Mahajan R.K. (2010). Classification of genetic diversity in abelmoschus tuberculatus germplasm collection using morphometric data. Ann. Appl. Biol..

[8] Oyelade O.J., Ade-Omowaye B.I.O., Adeomi V.F. (2003). Influence of variety on protein, fat contents and some physical characteristics of okra seeds. J. Food Eng..

[9] Anwar F., Rashid U., Ashraf M., Nadeem M. (2010). Okra (*Hibiscus esculentus*) seed oil for biodiesel production. Appl. Energy.

[10] Gemede H.F. (2015). Nutritional quality and health benefits of okra (*Abelmoschus esculentus*): A review. J. Food Process. Technol..

[11] Irshad M., He B.Z., Liu S., Mitra S., Debnath B., Li M., Rizwan H.M., Qiu D.L. (2017). In vitro regeneration of *Abelmoschus esculentus* L. Cv. Wufu: Influence of anti-browning additives on phenolic secretion and callus formation frequency in explants. Hortic. Environ. Biote.

[12] Anisuzzaman M., Kabir A.H., Sarker K.K., Jarin S., Alam M.F. (2010). Micropropagation of *Abelmoschus esculentus* L. (moench.) for disease free plantlets through meristem culture. Arch. Phytopathol. Plant Protect..

[13] Tester M., Davenport R. (2003). Na+ tolerance and na+ transport in higher plants. Ann. Bot.-Lond..

[14] Deinlein U., Stephan A.B., Horie T., Luo W., Xu G.H., Schroeder J.I. (2014). Plant salt-tolerance mechanisms. Trends Plant Sci..

[15] Bonhomme L., Valot B., Tardieu F., Zivy M. (2012). Phosphoproteome dynamics upon changes in plant water status reveal early events associated with rapid growth adjustment in maize leaves. Mol. Cell Proteom..

[16] Harb A., Krishnan A., Ambavaram M.M.R., Pereira A. (2010). Molecular and physiological analysis of drought stress in arabidopsis reveals early responses leading to acclimation in plant growth. Plant Physiol..

[17] Shi H.Z., Ishitani M., Kim C.S., Zhu J.K. (2000). The arabidopsis thaliana salt tolerance gene sos1 encodes a putative na+/h+ antiporter. Proc. Natl. Acad. Sci. USA.

[18] Qiu Q.S., Guo Y., Dietrich M.A., Schumaker K.S., Zhu J.K. (2002). Regulation of sos1, a plasma membrane na+/h+ exchanger in arabidopsis thaliana, by sos2 and sos3. Proc. Natl. Acad. Sci. USA.

[19] Berridge M.J., Lipp P., Bootman M.D. (2000). The versatility and universality of calcium signalling. Nat. Rev. Mol. Cell Biol..

[20] Sanchez-Barrena M.J., Fujii H., Angulo I., Martinez-Ripoll M., Zhu J.K., Albert A. (2007). The structure of the c-terminal domain of the protein kinase atsos2 bound to the calcium sensor atsos3. Mol. Cell.

[21] Quintero F.J., Ohta M., Shi H., Zhu J.K., Pardo J.M. (2002). Reconstitution in yeast of the arabidopsis sos signaling pathway for na+ homeostasis. Proc. Natl. Acad. Sci. USA.

[22] D’Autreaux B., Toledano M.B. (2007). Ros as signalling molecules: Mechanisms that generate specificity in ros homeostasis. Nat. Rev. Mol. Cell Biol..

[23] Finkel T. (2011). Signal transduction by reactive oxygen species. J. Cell Biol..

[24] Chen Y., Hoehenwarter W. (2015). Changes in the phosphoproteome and metabolome link early signaling events to rearrangement of photosynthesis and central metabolism in salinity and oxidative stress response in arabidopsis. Plant Physiol..

[25] Nakagami H., Kiegerl S., Hirt H. (2004). Omtk1, a novel mapkkk, channels oxidative stress signaling through direct mapk interaction. J. Biol. Chem..

[26] Mithoe S.C., Menke F.L.H. (2011). Phosphoproteomics perspective on plant signal transduction and tyrosine phosphorylation. Phytochemistry.

[27] Reinders J., Sickmann A. (2005). State-of-the-art in phosphoproteomics. Proteomics.

[28] Peck S.C., Nuhse T.S., Hess D., Iglesias A., Meins F., Boller T. (2001). Directed proteomics identifies a plant-specific protein rapidly phosphorylated in response to bacterial and fungal elicitors. Plant Cell.

[29] Xu X.W., Ji J., Ma X.T., Xu Q., Qi X.H., Chen X.H. (2016). Comparative proteomic analysis provides insight into the key proteins involved in cucumber (*Cucumis sativus* L.) adventitious root emergence under waterlogging stress. Front. Plant Sci..

[30] Xu D.B., Yuan H.W., Tong Y.F., Zhao L., Qiu L.L., Guo W.B., Shen C.J., Liu H.J., Yan D.L., Zheng B.S. (2017). Comparative proteomic analysis of the graft unions in hickory (carya cathayensis) provides insights into response mechanisms to grafting process. Front. Plant Sci..

[31] Hao J., Guo H., Shi X., Wang Y., Wan Q., Song Y.B., Zhang L., Dong M., Shen C. (2017). Comparative proteomic analyses of two taxus species (taxus x media and taxus mairei) reveals variations in the metabolisms associated with paclitaxel and other metabolites. Plant Cell Physiol..

[32] Lewandowska-Gnatowska E., Johnston M.L., Antoine W., Szczegielniak J., Muszynska G., Miernyk J.A. (2011). Using multiplex-staining to study changes in the maize leaf phosphoproteome in response to mechanical wounding. Phytochemistry.

[33] Xue L., Wang P.C., Wang L.S., Renzi E., Radivojac P., Tang H.X., Arnold R., Zhu J.K., Tao W.A. (2013). Quantitative measurement of phosphoproteome response to osmotic stress in arabidopsis based on library-assisted extracted ion chromatogram (laxic). Mol. Cell Proteom..

[34] Yang Z.B., Eticha D., Fuhrs H., Heintz D., Ayoub D., Van Dorsselaer A., Schlingmann B., Rao I.M., Braun H.P., Horst W.J. (2013). Proteomic and phosphoproteomic analysis of polyethylene glycol-induced osmotic stress in root tips of common bean (*Phaseolus vulgaris* L.). J. Exp. Bot..

[35] Zhang M., Lv D., Ge P., Bian Y., Chen G., Zhu G., Li X., Yan Y. (2014). Phosphoproteome analysis reveals new drought response and defense mechanisms of seedling leaves in bread wheat (*Triticum aestivum* L.). J. Proteom..

[36] Yu B., Li J.N., Koh J., Dufresne C., Yang N., Qi S.S., Zhang Y.X., Ma C.Q., Duong B.V., Chen S.X. (2016). Quantitative proteomics and phosphoproteomics of sugar beet monosomic addition line m14 in response to salt stress. J. Proteom..

[37] Hu X.L., Wu L.J., Zhao F.Y., Zhang D.Y., Li N.N., Zhu G.H., Li C.H., Wang W. (2015). Phosphoproteomic analysis of the response of maize leaves to drought, heat and their combination stress. Front. Plant Sci..

[38] Kline K.G., Barrett-Wilt G.A., Sussman M.R. (2010). In planta changes in protein phosphorylation induced by the plant hormone abscisic acid. Proc. Natl. Acad. Sci. USA.

[39] Khan M., Takasaki H., Komatsu S. (2005). Comprehensive phosphoproteome analysis in rice and identification of phosphoproteins responsive to different hormones/stresses. J. Proteome Res..

[40] Jones A.M.E., Bennett M.H., Mansfield J.W., Grant M. (2006). Analysis of the defence phosphoproteome of arabidopsis thaliana using differential mass tagging. Proteomics.

[41] Barjaktarovic Z., Schutz W., Madlung J., Fladerer C., Nordheim A., Hampp R. (2009). Changes in the effective gravitational field strength affect the state of phosphorylation of stress-related proteins in callus cultures of arabidopsis thaliana. J. Exp. Bot..

[42] Zieske L.R. (2006). A perspective on the use of itraq (tm) reagent technology for protein complex and profiling studies. J. Exp. Bot..

[43] Chen M., Zhu A., Storey K.B. (2016). Comparative phosphoproteomic analysis of intestinal phosphorylated proteins in active versus aestivating sea cucumbers. J. Proteom..

[44] Li Z., Li M., Li X., Xin J., Wang Y., Shen Q.W., Zhang D. (2018). Quantitative phosphoproteomic analysis among muscles of different color stability using tandem mass tag labeling. Food Chem..

[45] Wijk K.J.V., Schulze W.X. (2014). Meta-analysis of arabidopsis thaliana phospho-proteomics data reveals compartmentalization of phosphorylation motifs. Plant Cell.

[46] Yuan L.L., Zhang M., Yan X., Bian Y.W., Zhen S.M., Yan Y.M. (2016). Dynamic phosphoproteome analysis of seedling leaves in *Brachypodium distachyon* L. Reveals central phosphorylated proteins involved in the drought stress response. Sci. Rep..

[47] Heng Z., Bing H., Tai W., Sixue C., Haiying L., Yuhong Z., Shaojun D. (2012). Mechanisms of plant salt response: Insights from proteomics. J. Proteome Res..

[48] Lane T.S., Rempe C.S., Davitt J., Staton M.E., Peng Y., Soltis D.E., Melkonian M., Deyholos M., Leebens-Mack J.H., Chase M. (2016). Diversity of abc transporter genes across the plant kingdom and their potential utility in biotechnology. BMC Biotechnol..

[49] Verrier P.J., Bird D., Burla B., Dassa E., Forestier C., Geisler M., Klein M., Kolukisaoglu U., Lee Y., Martinoia E. (2008). Plant abc proteins--a unified nomenclature and updated inventory. Trends Plant Sci..

[50] Maeda H., Song W., Sage T., DellaPenna D. (2014). Role of callose synthases in transfer cell wall development in tocopherol deficient arabidopsis mutants. Front. Plant Sci..

[51] Sun H.G., Xia B.L., Wang X., Gao F., Zhou Y.J. (2017). Quantitative phosphoproteomic analysis provides insight into the response to short-term drought stress in ammopiptanthus mongolicus roots. Int. J. Mol. Sci..

[52] Fristedt R., Willig A., Granath P., Crevecoeur M., Rochaix J.D., Vener A.V. (2009). Phosphorylation of photosystem ii controls functional macroscopic folding of photosynthetic membranes in arabidopsis. Plant Cell.

[53] Vener A.V., Ohad I., Andersson B. (1998). Protein phosphorylation and redox sensing in chloroplast thylakoids. Curr. Opin. Plant Biol..

[54] Kitajima M., Butler W.L. (1975). Quenching of chlorophyll fluorescence and primary photochemistry in chloroplasts by dibromothymoquinone. Biochim. Biophys. Acta.

[55] Jonas S., Izaurralde E. (2013). The role of disordered protein regions in the assembly of decapping complexes and rnp granules. Gene Dev..

[56] Jiang Y., Yang B., Harris N.S., Deyholos M.K. (2007). Comparative proteomic analysis of nacl stress-responsive proteins in arabidopsis roots. J. Exp. Bot..

[57] Kawaguchi R., Girke T., Bray E.A., Bailey-Serres J. (2004). Differential mrna translation contributes to gene regulation under non-stress and dehydration stress conditions in arabidopsis Thaliana. Plant J..

[58] Deyholos M.K. (2010). Making the most of drought and salinity transcriptomics. Plant Cell Environ..

[59] Sunkar R., Li Y.F., Jagadeeswaran G. (2012). Functions of micrornas in plant stress responses. Trends Plant Sci..

[60] Christian Z.R., Sigrid S., Mühling K.H. (2010). Proteomic changes in maize roots after short-term adjustment to saline growth conditions. Proteomics.

[61] Dea-Wook K., Randeep R., Ganesh Kumar A., Young-Ho J., Junko S., Nam-Soo J., Yumiko I., Hitoshi I., Hyun K.D., Ie-Sung S. (2010). A hydroponic rice seedling culture model system for investigating proteome of salt stress in rice leaf. Electrophoresis.

[62] Mahfouz M.M., Kim S., Delauney A.J., Verma D.P. (2006). Arabidopsis target of rapamycin interacts with raptor, which regulates the activity of S6 kinase in response to osmotic stress signals. Plant Cell.

[63] Ruvinsky I., Meyuhas O. (2006). Ribosomal protein S6 phosphorylation: From protein synthesis to cell size. Trends Biochem. Sci..

[64] Feng X.P., Yu C.L., Chen Y., Peng J.Y., Ye L.H., Shen T.T., Wen H.Y., He Y. (2018). Non-destructive determination of shikimic acid concentration in transgenic maize exhibiting glyphosate tolerance using chlorophyll fluorescence and hyperspectral imaging. Front. Plant Sci..

[65] Zhang C.H., Dong W.Q., Gen W., Xu B.Y., Shen C.J., Yu C.L. (2018). De novo transcriptome assembly and characterization of the synthesis genes of bioactive constituents in *Abelmoschus esculentus* (L.) moench. Genes.

