# Supplementary figures and images for: The Phosphoproteomic Response of Okra (Abelmoschus esculentus L.) Seedlings to Salt Stress
Source: Int J Mol Sci. 2019 Mar 13;20(6):1262. doi: 10.3390/ijms20061262 (PMC6470868; doi:10.3390/ijms20061262)

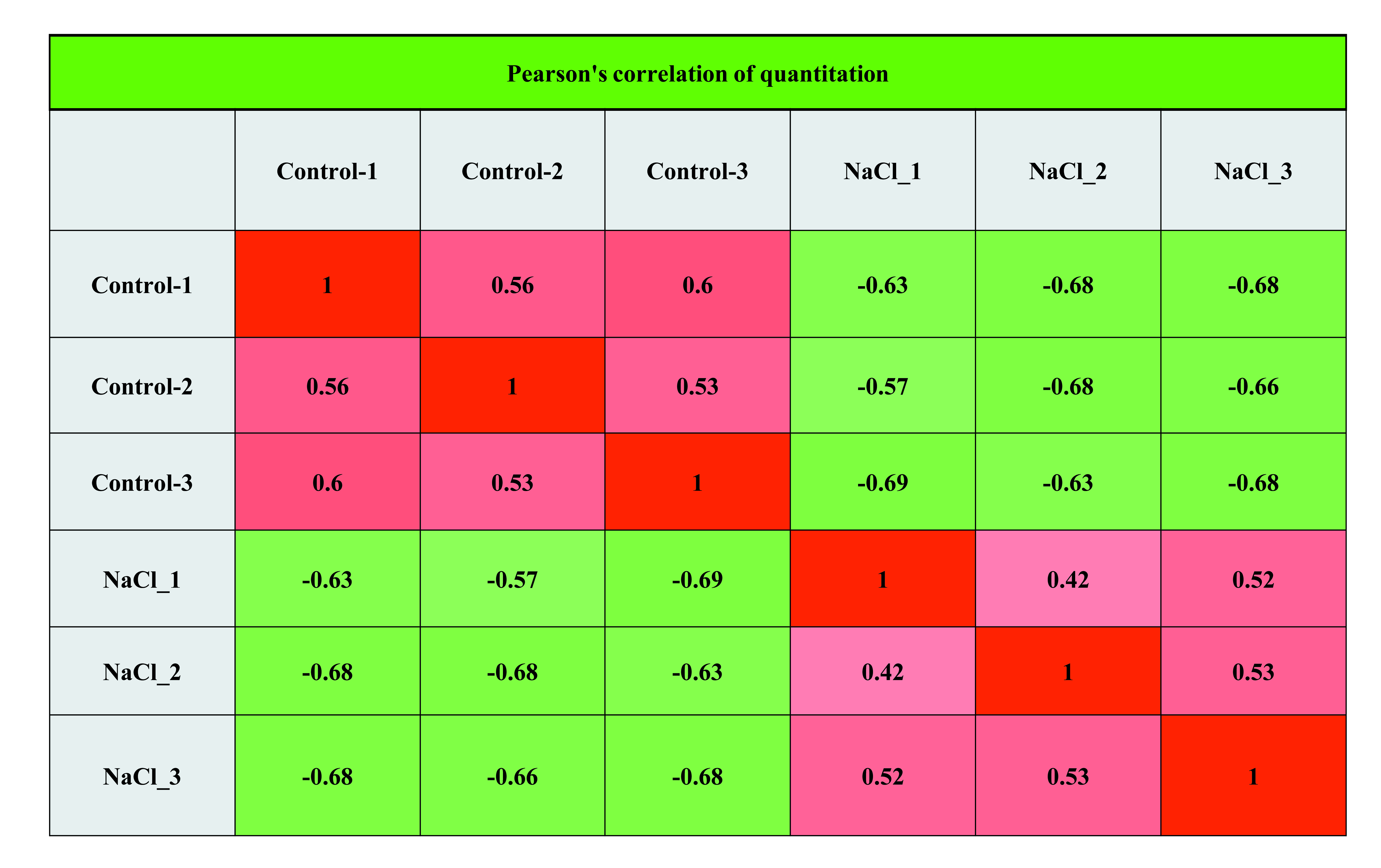

Supplement: Supplementary file 1 [file ijms-20-01262-s001.zip › Supplementary Materials/Figure S1.tif]

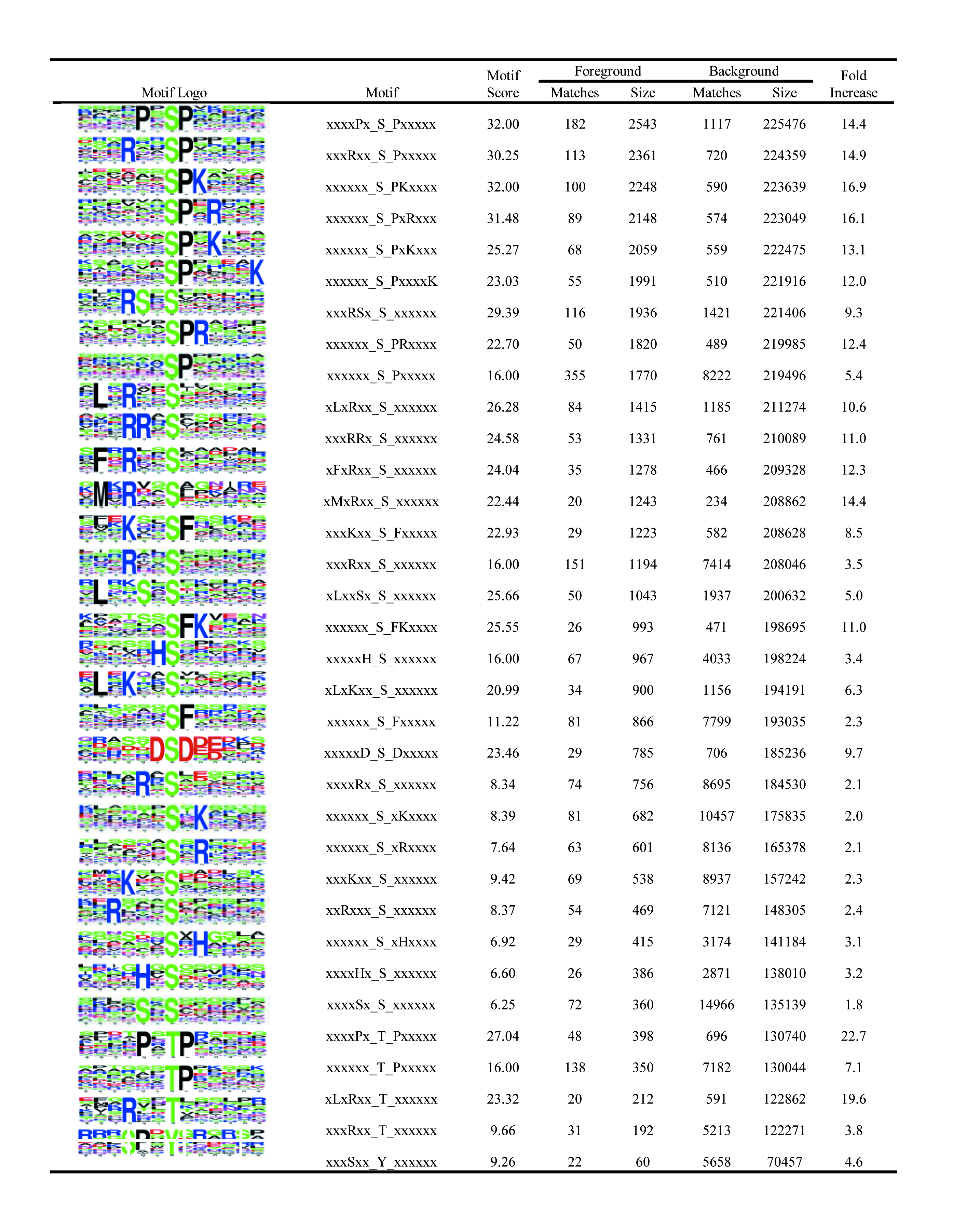

Supplement: Supplementary file 1 [file ijms-20-01262-s001.zip › Supplementary Materials/Figure S2.tif]

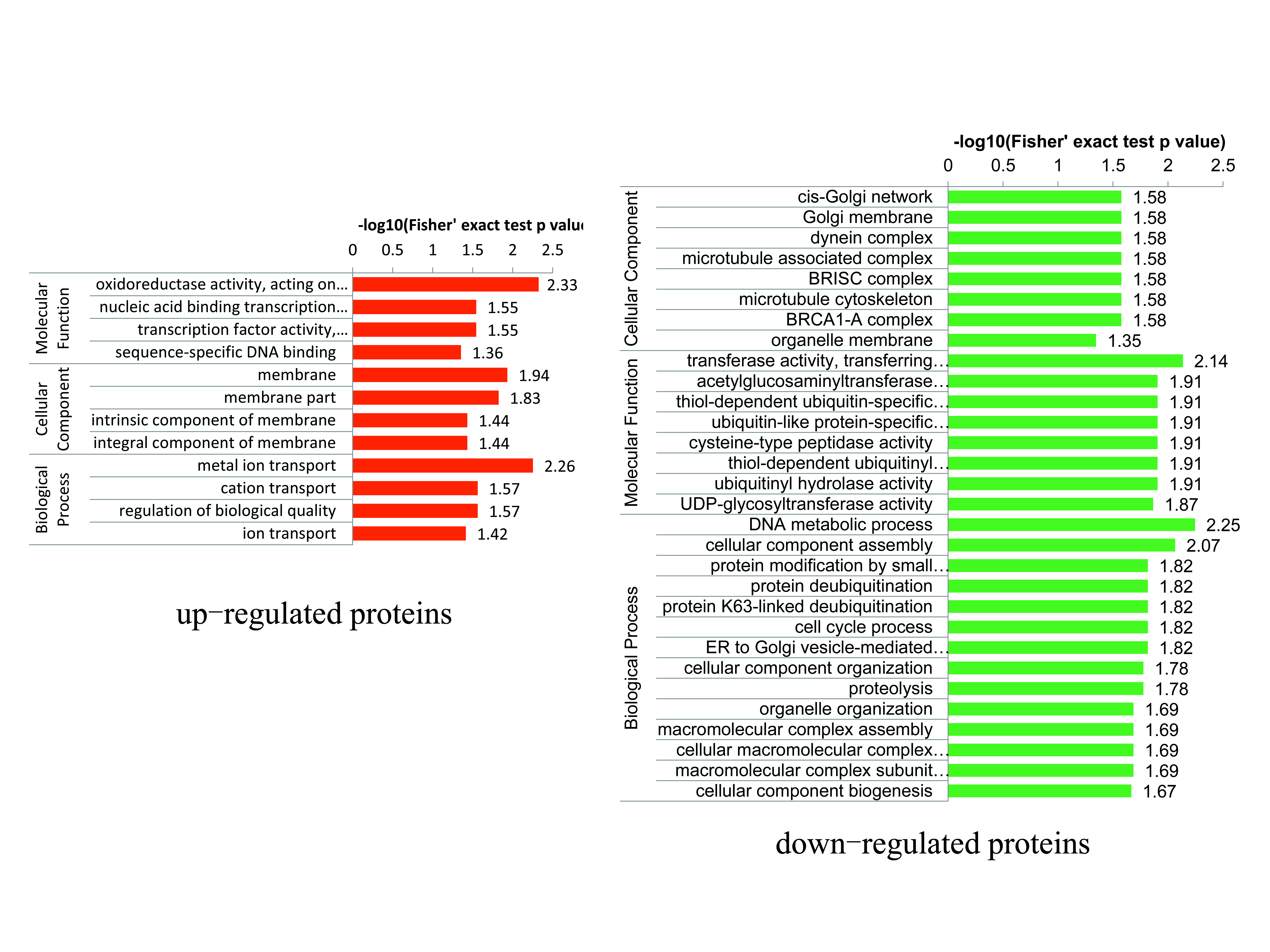

Supplement: Supplementary file 1 [file ijms-20-01262-s001.zip › Supplementary Materials/Figure S3.tif]
